# Improving Quality of ICD-10 (International Statistical Classification of Diseases, Tenth Revision) Coding Using AI: Protocol for a Crossover Randomized Controlled Trial

**DOI:** 10.2196/54593

**Published:** 2024-03-12

**Authors:** Taridzo Chomutare, Anastasios Lamproudis, Andrius Budrionis, Therese Olsen Svenning, Lill Irene Hind, Phuong Dinh Ngo, Karl Øyvind Mikalsen, Hercules Dalianis

**Affiliations:** 1 Health Data Analytics Norwegian Centre for E-health Research Tromsø Norway; 2 Department of Computer Science UiT The Arctic University of Norway Tromsø Norway; 3 Department of Physics and Technology UiT The Arctic University of Norway Tromsø Norway; 4 Clinic for Surgery, Oncology and Women Health University Hospital of North Norway Tromsø Norway; 5 The Norwegian Centre for Clinical Artificial Intelligence University Hospital of North Norway Tromsø Norway; 6 Department of Computer and Systems Sciences Stockholm University Kista Sweden

**Keywords:** International Classification of Diseases, Tenth Revision, ICD-10, International Classification of Diseases, Eleventh Revision, ICD-11, Easy-ICD, clinical coding, artificial intelligence, machine learning, deep learning

## Abstract

**Background:**

Computer-assisted clinical coding (CAC) tools are designed to help clinical coders assign standardized codes, such as the *ICD-10* (*International Statistical Classification of Diseases, Tenth Revision*), to clinical texts, such as discharge summaries. Maintaining the integrity of these standardized codes is important both for the functioning of health systems and for ensuring data used for secondary purposes are of high quality. Clinical coding is an error-prone cumbersome task, and the complexity of modern classification systems such as the *ICD-11* (*International Classification of Diseases, Eleventh Revision*) presents significant barriers to implementation. To date, there have only been a few user studies; therefore, our understanding is still limited regarding the role CAC systems can play in reducing the burden of coding and improving the overall quality of coding.

**Objective:**

The objective of the user study is to generate both qualitative and quantitative data for measuring the usefulness of a CAC system, Easy-ICD, that was developed for recommending *ICD-10* codes. Specifically, our goal is to assess whether our tool can reduce the burden on clinical coders and also improve coding quality.

**Methods:**

The user study is based on a crossover randomized controlled trial study design, where we measure the performance of clinical coders when they use our CAC tool versus when they do not. Performance is measured by the time it takes them to assign codes to both simple and complex clinical texts as well as the coding quality, that is, the accuracy of code assignment.

**Results:**

We expect the study to provide us with a measurement of the effectiveness of the CAC system compared to manual coding processes, both in terms of time use and coding quality. Positive outcomes from this study will imply that CAC tools hold the potential to reduce the burden on health care staff and will have major implications for the adoption of artificial intelligence–based CAC innovations to improve coding practice. Expected results to be published summer 2024.

**Conclusions:**

The planned user study promises a greater understanding of the impact CAC systems might have on clinical coding in real-life settings, especially with regard to coding time and quality. Further, the study may add new insights on how to meaningfully exploit current clinical text mining capabilities, with a view to reducing the burden on clinical coders, thus lowering the barriers and paving a more sustainable path to the adoption of modern coding systems, such as the new *ICD-11*.

**Trial Registration:**

clinicaltrials.gov NCT06286865; https://clinicaltrials.gov/study/NCT06286865

**International Registered Report Identifier (IRRID):**

DERR1-10.2196/54593

## Introduction

### Background

Artificial intelligence shows a lot of promise for many health care applications, but its implementation in clinical settings is still limited [1]. One promising implementation area is clinical coding, which involves clinical staff or trained personnel going through large amounts of clinical text, such as discharge summaries, and assigning clinical codes to the texts. Clinical staff can thus summarize the patient’s stay at the hospital using a system of codes such as the International Statistical Classification of Diseases and Related Health Problems (version 10; *ICD-10*[ *International Statistical Classification of Diseases, Tenth Revision*]) [2].

A systematic way of summarizing patient care at hospitals is important for multiple reasons. The primary use of such data is to judge both the quality and volume of care. The data also serve as a consideration point for resource allocation. In addition, good coding practice is important for improving the quality of data available for secondary purposes such as research and knowledge generation.

An analysis of the literature reveals at least 2 major uses for computer-assisted clinical coding (CAC) systems [3]. The first type of use is to assist clinical coders in their work in real time, while the other type of use is in auditing the quality of existing coding. Audits are a principal element of quality control, especially since there have been reports of significant coding errors in electronic health record (EHR) systems. For instance, in a Swedish study, Jacobsson and Serdén [4] reported that 20% of the main diagnosis codes were wrong. For this user study, we focus on the former type of use, that is, assisting clinical coders in real time.

### Available Knowledge

In terms of the informatics methods that CAC systems use, there is wide variation. Depending on specific use cases, rule-based methods have been successfully used to extract clinical concepts from texts [5-7] or to classify clinical texts into standard terminology such as Systematized Nomenclature of Medicine—Clinical Terms or International Classification of Diseases [8,9]. Rule-based methods appear to work well, but generally, texts have to be tokenized into short phrases or n-grams. Words within each sentence are usually treated independently of each other. Unless considered separately, clinical language peculiarities such as negation [10], uncertainty and speculations, and acronyms and abbreviations [11] can obscure the meaning of words in sentences.

With the advances in deep learning, in particular, transformer architectures, longer text sequences can be processed using contextual embeddings, where the representation of each word is based on its context. This contextual understanding of words makes deep learning an appropriate tool for CAC systems that process long and complex clinical texts. Santos et al’s [12] systematic review on the topic shows a clear timeline transition toward deep learning methods, and this finding is echoed in a recent review by Kaur et al [13]. However, occasioned by multiple limiting factors related to the nature of clinical coding, current state-of-the-art (SOTA) performance is still unsatisfactory and lags other application areas of deep learning.

One of the major limiting factors is that assigning clinical codes to large sequences of text is a hard problem, compounded by a very large number of codes that represent the label space (eg, more than 20,000 *ICD-10* codes for Norwegian). In addition to the large number of codes, the level of class imbalance is rather extreme in this use case. Another limiting factor is that many close codes have semantically similar descriptions. An example of 2 semantically similar codes is “Other diseases of tongue (K14.8)” and “Disease of tongue, unspecified (K14.9).” It is challenging for a model to discriminate the two, since they both appear to describe some unnamed condition of the tongue [14]. Combined with other limiting factors such as poor data quality and availability, these factors make the design of automatic *ICD-10* coding systems nearly unattainable, especially for minor languages such as Norwegian and Swedish.

To get around this automatic coding problem, 2 popular approaches have emerged. In the first approach, researchers experiment with just a small portion of the codes, for instance, top-10 [15] or top-50 codes [16]. In many instances, these few top-N codes represent the majority of the data, but such systems perform poorly on rare codes. In the second approach, rather than automatically assigning *ICD-10* codes, multiple possible codes are provided as suggestions, much like recommender systems, with top-N suggestions. It is conceivable that this second work-around approach is useful to clinical coders because it provides potentially meaningful cues and pointers to the correct codes. This is the approach taken by the current user study.

It is important to note that a number of inconsistent findings have emerged from the little that has been published. In a recent study, Chen et al [17] concluded that, while their system improved the accuracy of *ICD-10* assignment, the system did not reduce the time required to assign the codes. In complete contrast to these findings, an earlier study by Wang et al [18] found significant time efficiencies when the CAC system was used, as did Fung et al [19]. We hypothesize that text complexity partially explains the conflict. Since text complexity is a factor that has not been fully explored in existing studies, we base our analysis of accuracy and time on the complexity of the clinical text.

### Specific Aims

The aim of the project is to investigate artificial intelligence tools, natural language processing specifically, to improve the quality of *ICD-10* coding. The goal of the user study is to test whether our Easy-ICD system can reduce the burden of coding and also improve the quality of *ICD-10* coding. The main end points to measure are (1) the individual’s coding accuracy averaged across all instances and *F*_1_-score (harmonic mean of recall and precision, each of which is averaged over all instances), (2) time use for assigning codes, and (3) usability based on the system usability scale [20].

## Methods

### Study Population and Location

The user study will recruit clinical coders (physicians, health care staff, professional coders, etc) from Norway and Sweden on a rolling basis. Even though clinical notes are in Swedish, most of the target population understands both the Scandinavian languages.

### Inclusion and Exclusion Criteria

In terms of participants, the main inclusion criterion is that the participant has coded clinical texts before, preferably *ICD-10* coding. Participants could be clinicians, nurses, professional coders, or other health care staff who understand Swedish with any amount of coding experience. We shall exclude participants outside Sweden or Norway.

### Study Time Frame

Recruitment of participants commenced toward the end of 2023, and we will continue recruitment on a rolling basis until the target number of participants is reached.

### Ethical Considerations

The data curated for this study is available through Permission Dnr 2022-02386-02 from the Swedish Ethical Review Authority. This user study is part of the ClinCode Project approved by The Regional Committee for Medical and Health Research Ethics, Norway (260972). Participation is based on informed consent. A web page with information based on the template from the Norwegian Centre for Research Data is used to provide information about (1) purpose of the project; (2) institution responsible for the project; (3) why the user is being asked to participate; (4) what the user is expected to do as a participant; (5) participation as voluntary; (6) user personal privacy, when and what happens with their data; (7) information about user rights (under the General Data Protection Regulation); (8) who to contact for more information or concerns; and (9) the final section where the user clicks on “Consent” or “Decline.” If the user consents to participate, the consent is logged, and the user can proceed to the study. Thus, we have a written record of consent. If the user declines, they are redirected away from the study. It is important to note that no personal or identifying information is collected, and browser cookies are only used to track progress. Thus, all the data we store will be completely anonymous.

### Intervention

The CAC system, Easy-ICD, is based on deep learning transformer models, also called language models. The system uses a language model trained in 3 main cycles, based on a typical natural language processing pipeline involving unsupervised or semisupervised training (pretraining) and supervised training (fine-tuning). First, the base model, Kungliga Biblioteket—Bidirectional Encoder Representations From Transformers (KB-BERT), was obtained from the transformers library [21]. KB-BERT is a Swedish general language model that was pretrained by the National Library of Sweden [22] and is publicly available. This pretraining represents the first training cycle. In the second cycle, KB-BERT was further pretrained on 17.8 GB of pseudonymized Swedish clinical text from the Health Bank infrastructure at Stockholm University [11], resulting in the current clinical language model named SweDeClinBERT [23]. In the final training cycle, also called fine-tuning, the model was fine-tuned on a pseudonymized data set, the Stockholm EPR GastroICD-10 Pseudo Corpus II, encompassing 120,000 patients. Using pseudonymized data for training improves security by decreasing the likelihood of private information leakage.

This model achieves SOTA results on this clinical coding task, comparable to results reported for English health data sets like the Medical Information Mart for Intensive Care [24]. This clinical coding task is framed as a multilabel problem, where participants can select and assign more than one *ICD-10* code to the clinical text. Actual performance results of the model are reported in a paper under consideration, and these results will be released in the final reporting of the user study.

Considering the Easy-ICD user interface, instead of displaying only the top label or labels as predicted by the model, we display the top-N predictions, where N is between 5 and 10. Through experimentation, we determined that an ensemble output from the deep learning model and fuzzy logic had the best results. Fuzzy logic is based on word-level and sentence-level minimum edit distance, using Levenshtein distance [25], comparing between *ICD-10* descriptions and the clinical text.

Easy-ICD is implemented as a web application for this user study, but in the future, the functionality will be incorporated into an EHR system. We used the classical model-view-controller pattern involving the deep learning model module, HTML 5 web interface, and a Python back-end controller, respectively. The web interface has the following sections: (1) the clinical note, (2) color-coded visualization to explain the suggestions, (3) the top-N suggestions from where the participant selects relevant *ICD-10* codes to assign, (4) qualitative feedback with star-rating and text feedback, and (5) a lookup feature for *ICD-10* codes.

### Disease and Data

We use Swedish clinical notes in the gastrointestinal (gastro) domain in our study. The data used are from the gastrointestinal department of the Health Bank infrastructure at Stockholm University [11]. The data contain both in- and outpatient records of patients receiving treatment at the department, both surgery and other medical interventions. The data originate from a hospital, and no effort was made to include only similar cases; therefore, the cases vary.

The data are pseudonymized and have been curated by 2 professional clinical coders, so as to double-check the coding and create a gold standard. The 2 coders reviewed the data independently and reconciled their differences through discussion. This curation process also ensures that it will be possible to assign a code based on the clinical text alone, without the need for further information about the patient. This is important since the user study will not have access to further EHR data such as laboratory results or drug lists.

### Study Design

**Figure 1:**
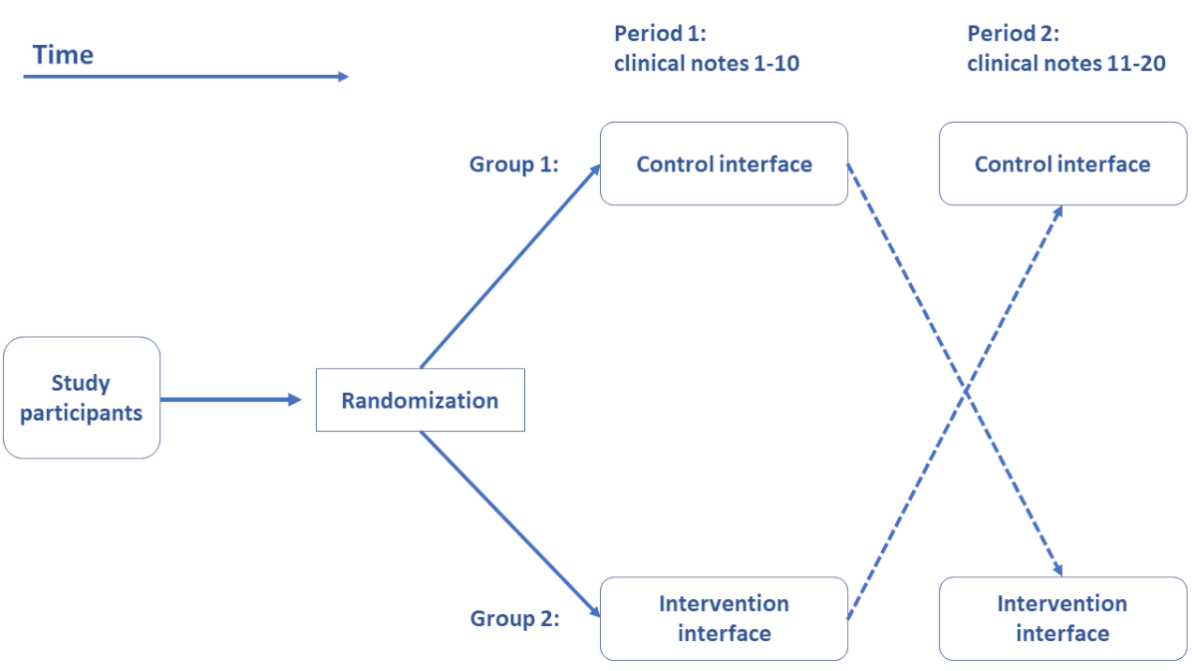
Crossover randomized controlled trial where study participants are randomized into 2 groups. The first group first uses a control interface and then switches to our Easy-ICD tool, while the second group starts with our tool and switches to a control interface.

This study is designed as a 2×2 crossover study as illustrated in Figure 1. This design, AB|BA (2 different sequences and 2 periods), is chosen for its uniform and balanced properties, aiming to control the carryover effect between periods. The design is suitable not only for comparing treatments but also for assessing pre-post effect of an intervention [26]. In addition, the crossover design requires a comparatively smaller number of participants than what a randomized controlled trial would ordinarily require.

We will not incorporate a washout period partly because the learning element in the tasks measured is limited and partly because we do not want to risk losing participants in a long break between periods. The data sets in the 2 periods are different, and this limits any carryover effect in the sense that coders cannot memorize the text or the correct codes.

Once participants are recruited, they are randomly allocated to the 2 groups without allocation concealment. Allocation concealment will not be relevant for coders since it is known whether a participant is assisted or not, and we will not develop a placebo coding assistant. We will, however, conceal the allocation of subjects for the analyses.

In total, participants will code 20 clinical notes, where each note belongs to a single patient. The participants are asked to complete the experiment in 1 sitting without interruptions, and they cannot revisit or go back to previous notes. In the event that participants are interrupted, they are asked to exit the experiment, and any incomplete experiments are discarded as invalid.

In terms of the coding process, both the control and intervention user interfaces have a search utility for looking up *ICD-10* codes. Participants will be free to access any other additional search tools they wish to use without restriction. Since we measure time using a before and after design, the effect of additional tools is not expected to be a significant factor. The time is logged based on the browser button presses such as “Start,” “Next,” and “Complete.”

The user study process can be summarized in the following steps:

1. Study participants are randomly allocated to group 1 and group 2.
2. To prepare participants for the experiment, a short video tutorial is played after the consent form is signed and right before the clinical coding task commences.
3. In period 1 with 10 clinical notes, group 1 uses the control interface, while group 2 uses the intervention interface.
4. Data are logged in the background using button presses—time, assigned codes, and comments.
5. Then, there is an immediate crossover to period 2 for the last 10 clinical notes.
6. Data continue to be logged in the background using button presses.
7. At the end, participants in both groups will complete the system usability scale.

### Sample Size Calculation

**Figure 2:**
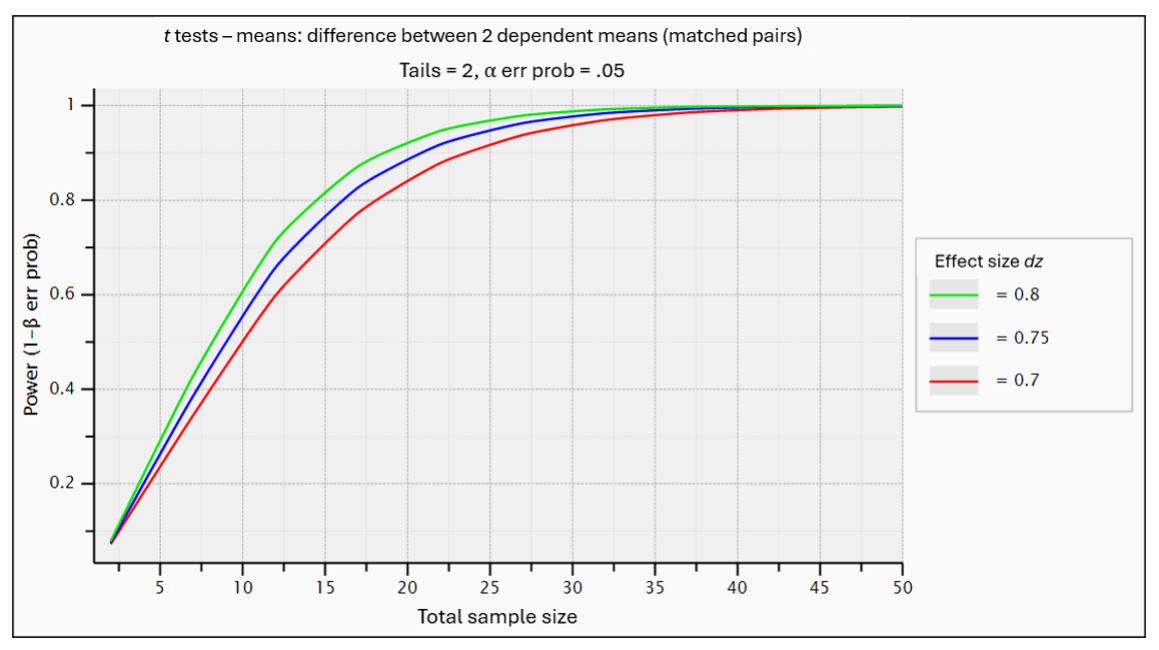
Statistical power of a 2-tailed t test at the level of 5% depending on sample size per sequence (2-tailed t test with equal SD in both study sequences to compare means).

This experiment is novel, and we have not succeeded in finding many similar studies from which to estimate test parameters. Therefore, our experiment is important in that it provides baselines that may be useful for similar experiments in the future. We do expect a correlation between periods; the same coders are measured twice on mean performance in time, making a matched 2-tailed *t* test suitable for the task [26]. This matched 2-tailed *t* test (period) is done in 2 sequences, and the sample size calculation must therefore be doubled [27]. Further, each sequence will consist of approximately 10 coding instances, giving extra power to the estimation of the sequence mean. Due to the unknown nature of the expected effect size, the sample calculation in Figure 2 is made with 3 different effect estimates ranging in the upper level of medium to high effect. A pilot study is necessary to establish a probable effect size and adjust sample size estimation if needed.

Based on the plot in Figure 2 derived from power analysis in G*Power [28], we aim to recruit up to 30 participants in total. Multiple methods are used to recruit participants, including contacting hospitals and health authorities directly, announcing at coding seminars and conferences, publishing news in popular science media, and advertising on social media groups and other electronic media.

### Measurements and Analyses

The focus of our measurements is on how clinical coders perform with and without our system:

1. Compared with the performance of the unaided user, statistical tests of significance are used to check whether there are significant differences in the quality of coding when Easy-ICD is used, as measured by accuracy and *F*_1_-score.
2. Time spent is the time it takes for a clinical coder to assign a code.
3. System usability survey will be given to all participants to measure usability of the web interfaces, both with and without our Easy-ICD system.

Main analyses are based on hypothesis testing using the paired *t* test and Wilcoxon test to compare the before and after means at a significance level of .05. Table 1 summarizes these hypotheses based on a test of equivalence.

**Table 1:** Time use and coding quality hypotheses testing to compare the before and after means.

Time use		Coding quality (accuracy and *F*_1_-score)
**Null hypothesis: (H_0_: μ_1_=μ_2_)**		
	There is no time difference between using CAC^a^ and not using CAC for coding Swedish clinical notes	CAC does not affect a clinical coder’s coding quality
**Alternative hypothesis: (H_1_: μ_1_≠μ_2_)**		
	There is a significant time difference between using CAC and not using CAC for coding Swedish clinical notes	CAC affects a clinical coder’s coding quality by a significant margin

^a^CAC: computer-assisted clinical coding.

In additional exploratory analyses, we will examine the effect wrong suggestions have on the coder’s performance, and how often coders adopt these wrong suggestions. In addition, the distribution of the different professions between the groups as well as the coding experience will be explored.

To enable us to analyze the main outcomes in relation to text complexity, we distinguish between two categories of clinical texts: (1) short and simple texts (<512 tokens) and (2) long and complex texts (>512 tokens). However, we only consider the length of the text as the complexity dimension, as opposed to the complexity of the case or provided care, for example.

## Results

### Overview

Our results will be based on the statistical significance of the 2 tests related to time use and coding quality, which will allow us to conclude whether or not our CAC tool, the Easy-ICD, has the potential to impact clinical coding practice in a meaningful way. The expected results to be published summer 2024.

### Evaluation Outcomes

The evaluation outcomes are as follows: (1) clinical coders aided by our Easy-ICD system are expected to yield better performance, that is, the accuracy and *F*_1_-score. (2) We expect that the Easy-ICD system will result in time savings during coding tasks. Looking at the 2 categories of clinical notes, it can be expected that the most time savings could be gained with large and complex texts. Such cases take more resources than shorter and simpler texts. (3) We expect the usability of the system to have a system usability score over the generally accepted normal score of 68 [29]. Further, we do not expect any significant differences between the usability of the user interfaces with and without our CAC system.

## Discussion

### Principal Findings

There are 2 primary areas of interpretation with which our study is concerned. The first relates to the quality or accuracy of clinical coding. If it shows that coders aided by our system perform better than unaided coders, we can conclude that our system is a useful tool to improve the quality of clinical coding. If, on the other hand, no significant improvement in accuracy is noted, we have to look to the other measurement point for complete interpretation. Time efficiency is the other interpretation point, where we would like to see significant time improvement between the intervention and control.

If the accuracy is not better, then the time savings must be significant. If there are no time savings, then the improvement in accuracy must be significant. It can also be argued that the CAC system is still useful even if the timing is slightly worse, while the performance is better by a significant margin.

If neither time nor accuracy improves, then we have to think about improving the system for future studies. Regardless, we plan to publish our findings in an international (or Nordic) peer-reviewed journal or conference and note all the facilitating factors and barriers.

In terms of usability, if there are no significant differences in usability scores, then usability issues can be ruled out. If the CAC system has significantly better usability scores, we expect the usability scores of the standard interface to be no worse than what is considered normal.

### Limitations

Perhaps one limitation of this study is that we curate the clinical texts used in the study so that the participants should be able to assign a code simply by assessing the text. In practice, some clinical texts need supporting information from other information sources such as pathology or radiology systems [9]. This supporting information would normally be available in patient record systems.

Another limitation is that we only considered data from the gastrointestinal department (K-codes), and this *ICD-10* chapter has a comparatively smaller number of codes. There were 415 unique codes in the training data, and just over 480 K-codes exist in the Norwegian version of *ICD-10*. In contrast, the whole *ICD-10* system has a total of over 20,000 codes in Norwegian. Therefore, the generalizability of our system is an important factor to consider for future studies when expanding to other clinical domains.

### Comparison to Prior Work

Even though there are many papers on experimental work dealing with assigning *ICD-10* codes to clinical text, only a few studies with health care staff exist. The planned user study holds the potential to yield new insights about the usefulness of CAC systems in terms of reducing coding time and improving coding quality, both of which are important impact indicators for reducing the burden on clinical coders.

### Conclusions

In terms of impact, positive outcomes from this study contribute to the evidence that supports the adoption of CAC tools to reduce barriers to the implementation of modern coding systems such as the *ICD-11* (*International Classification of Diseases, Eleventh Revision*).

## Abbreviations

CAC: computer-assisted clinical coding
EHR: electronic health record
ICD-10: International Classification of Diseases, Tenth Revision
ICD-11: International Classification of Diseases, Eleventh Revision
KB-BERT: Kungliga Biblioteket—Bidirectional Encoder Representations From Transformers
SOTA: state-of-the-art

## Data Availability

Data sharing is not applicable to this paper as no data sets were generated or analyzed during this study.
